# Effective Boundary Correction for Deterministic Lateral Displacement Microchannels to Improve Cell Separation: A Numerical and Experimental Study

**DOI:** 10.3390/bios14100466

**Published:** 2024-09-29

**Authors:** Shaghayegh Mirhosseini, Mohammadmahdi Eskandarisani, Aryanaz Faghih Nasiri, Fatemeh Khatami, Akram Mirzaei, Majid Badieirostami, Seyed Mohammad Kazem Aghamir, Mohammadreza Kolahdouz

**Affiliations:** 1 School of Electrical and Computer Engineering, College of Engineering, University of Tehran, Tehran 1439957131, Iran; ppk9da@virginia.edu (S.M.); aryanaznasiri@ut.ac.ir (A.F.N.); mbadiei@ut.ac.ir (M.B.); 2 Department of Electrical and Computer Engineering, University of Virginia, Charlottesville, VA 22908, USA; 3 School of Mechanical Engineering, College of Engineering, University of Tehran, Tehran 1439957131, Iran; moe39@pitt.edu; 4 Department of Bioengineering, University of Pittsburgh, Pittsburgh, PA 15260, USA; 5 Urology Research Center, Tehran University of Medical Sciences, Tehran 1416753955, Iran; fatemehkhatami1978@gmail.com (F.K.); mirzaee.scholar@gmail.com (A.M.)

**Keywords:** microfluidics, deterministic lateral displacement, boundary correction, cell separation

## Abstract

Particle separation and sorting techniques based on microfluidics have found extensive applications and are increasingly gaining prominence. This research presents the design and fabrication of a microfluidic device for separating cells using deterministic lateral displacement (DLD), enabling accuracy and continuity while being size-based. Nevertheless, it remains demanding, to completely reverse the detrimental effects of the boundaries that disturb the fluidic flow in the channel and reduce particle separation efficiency. This study introduces a novel approach to enhance the boundary structure of channels. By using this design, separation efficiency is boosted, and the fluid behavior around the walls is improved. The boundary correction (BC) enhances the operation of the microchannel and is very effective in microchannels. With boundary correction, the device exhibited improved separation efficiencies, but in its absence, separation efficiencies dropped. The collected microscopic images of the isolation of prostate cancer cell lines and red blood cells revealed promising outcomes. The efficiency of circulating tumor cell (CTC) throughput in the microfluidic channel, quantified as the ratio or proportion of tumor cells exiting the channel to cells entering it, exceeds 93%. Moreover, the efficiency of CTC isolation, expressed as the proportion of tumor cells from the upper outlet of the microfluidic channel to all cells, is over 89%. Additionally, the efficiency of red blood cell isolation, evaluated as the ratio of red blood cells from the lower outlet of the microfluidic channel to all cells, surpasses 77%. While using the same DLD separator without boundary correction reduced the separation efficiency by around 5%.

## 1. Introduction

Microfluidic devices are miniaturized systems that can regulate the flow of liquids, suspended cells, and particles with high accuracy while continuously using only a small amount of a sample volume [1]. The separation of certain bioparticles within a sample is one of the most frequently used microfluidic capabilities for lab-on-a-chip technology [2]. This application makes the development of effective treatments and the diagnosis of many ailments possible. Particle analysis and separation techniques are two crucial phases that must be quick, inexpensive, effective, and continuous [3,4,5,6,7,8,9,10,11,12]. Numerous biomedical and biological experiments need the separation of cells; hence, various microfluidic separation techniques have been developed in response [13,14]. Microfluidics is frequently used for this purpose and makes separating target particles from biofluids like blood and urine with low concentrations possible [15]. In microfluidic systems, the separation of bioparticles mostly depends on the particles’ physical characteristics, for example, mass, size, electrical positivity or negativity, etc., and usually falls among active and passive types [16,17,18,19,20]. Microfluidics that sorts the particles and the flow using external fields is used as an active sorter. Dielectrophoresis, electrophoresis, magnetophoresis, thermophoresis, and acoustophoresis are a few active separation techniques. By applying non-uniform electric fields to the channel, dielectrophoresis separates particles according to their size and capacitance [21,22,23,24]. For particles to be segregated in the channel according to differences in their size, concentration, and the degree to which they can be compressed, acoustophoresis transmits bulk or ultrasonic surface waves [25,26,27,28,29,30]. Particles can be sorted by magnetophoresis, either intrinsically or super-paramagnetically, according to their magnetic susceptibilities [31,32,33,34,35,36]. On the other hand, passive systems, which offer comparable ease of use and output to the aforementioned method, primarily rely on the channel’s shape and hydrodynamic forces for separation [37]. Among the passive methods are deterministic lateral displacement (DLD) [38], pinched-flow fractionation (PFF), and hydrodynamic filtration [17,18,39,40,41,42].

Huang et al. first described the working principle of DLD in 2004, which classifies particles according to their diameters [43]. Particles are separated in a DLD device by a flow profile produced by an exact post array arrangement. This technique may be used to separate different components [44,45,46,47,48,49] of the blood like red blood cells (RBCs) [50,51,52], white blood cells (WBCs) [53], platelets (PLTs) [54], circulating tumor cells (CTCs) [55,56,57], spores [58], bacteria [59], parasites [60], and nanoparticles [61,62]. Exosomes [63] and DNA [64] are two other bioparticle types separated using DLD microfluidics. The DLD is cheaper, user-friendlier, more precise, and better at preserving the particle’s shape and characteristics compared to the previous approaches. It also offers the highest resolution among all the separation techniques [43]. DLD is capable of separating particles with diameters ranging from nanometers to millimeters. Other benefits reaping from employing DLD include label-free separation and an easy production procedure [65].

The shape of the channel is crucial to the DLD approach. The primary factor in separation is how the post arrays are designed and arranged. Migration angle (θ) refers to the angle formed by post arrays with respect to the flow direction. The spacing (G) between each pair of posts is also constant. A critical size (D_c_) for separation is determined by the gap (G) and migration angle (θ). In a DLD array, the zigzag and bump modes are the two primary particle movement modes. Particles will either follow the zigzag streamline or the bumping streamline [43,66]. Smaller-diameter particles compared to the D_c_ will experience the zigzag mode with minimal lateral displacement. On the contrary, cells with a diameter greater than the critical diameter D_c_ follow a bumping trajectory and move in the direction of the channel’s slope [43,66]. Particle sorting will be based on whether their sizes are either smaller or bigger than the critical diameter (D_c_). The switch from zigzag to bump mode occurs at a critical size. Most of the time, DLD separation is binary, meaning that there is only one critical diameter in a DLD array.

Numerous devices have been designed, simulated, and tested since the DLD principle was first introduced and explained, including those for filtering satellite droplets produced by microfluidic droplet generators [41,67,68,69], circulating tumor cell separation, and blood fractionation [70,71]. Other studies claimed that a DLD device might experience several separations. PLTs were initially isolated from a blood sample by Li et al. The remaining RBCs and WBCs were then separated using a different array with varied critical sizes [72]. In a DLD device with two stages, Pariset et al. isolated the E. coli bacteria, RBCs, and prostate cancer cells [73]. They also developed a droplet production approach to maintain pressure control when joining two stages of DLD arrays for multiple particle separations in a biological sample. These designs include at least two stages of DLD arrays, and each step separates a specific particle [73,74].

Cell separation by DLD microchannels requires standard photolithography methods. Polydimethylsiloxane (PDMS) is one of the most common materials for microfluidic devices; hence, PDMS-based devices are also quite popular [75]. The potential to quickly produce microfluidic devices from a master mold is one of the several appealing features that PDMS provides, along with biocompatibility, transparency, and the simplicity of device fabrication. However, particle blockage close to the boundary interfaces is one of the difficulties with the DLD approach. The posts in the array would have a unique Dc, and those at the boundaries would malfunction, i.e., when the edges remain unmodified, the post gaps deviate from the middle of the channel order, as the theory of DLD channel suggests. Clearly, the critical diameter (Dc) near the channel edge plays a crucial role in this deviation. Irregularities and anomalies in streamline patterns become apparent in these zones, hindering the expected periodic flow patterns, which as a result decreases or eliminates the separation effect, resulting in a failure in the anticipated yield of the streamlines and Dc as predicted by the theory.

In addition to conventional microfluidic separation techniques, recent advancements in microfluidic cell sorting have shown significant promise in improving the efficiency and accuracy of particle separation. Innovative systems, such as the hybrid platform for live/dead bacteria sorting using on-chip dielectrophoresis (DEP), have demonstrated high precision in distinguishing between viable and non-viable cells [76]. Similarly, the application of electrokinetic deterministic lateral displacement has been explored for efficient cell sorting. These developments underscore the potential of microfluidic technologies in achieving high-throughput and precise cell separation [77]. Also, another hybrid platform technique is filtration, along with inertial microfluidics and viscoelastic methods, which are all passive techniques. In contrast, active techniques include dielectrophoresis and acoustophoresis. These methods are commonly used for sorting red blood cells (RBCs) and white blood cells (WBCs) [78].

Herein, we propose a DLD sorter with a more effective wall-correction approach. The precise DLD sorter with circular posts separates CTC from blood cells to achieve highly pure target cells. With the boundary correction (BC), streamlines, and velocity contour, we regularized the flow similar to the flow in the middle of the microchannel, establishing the balance between the drag and lift forces. Using this technique, the separation efficiency can be increased while preventing the clogging of the particles.

## 2. Materials and Methods

### 2.1. The DLD Theory

**Figure 1:**
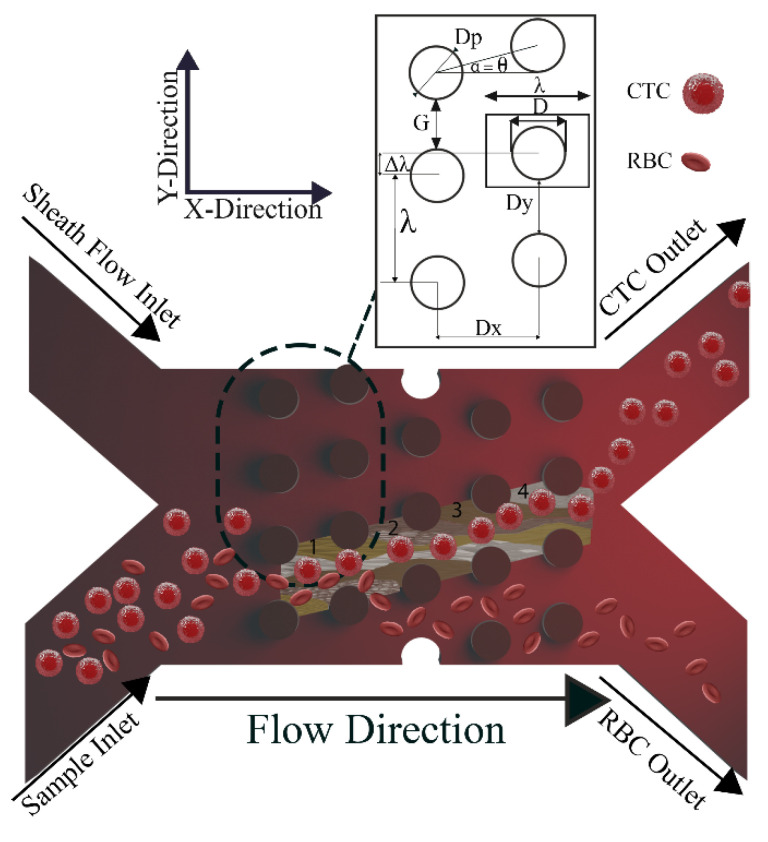
Top view schematic of the parameters in DLD sorter. The array of the DLD device with *n* = 4 columns represents the geometry and the working principle. Larger particles follow the posts’ path (bump/displacement mode) in a DLD array, whereas smaller particles follow the fluid’s path (zigzag mode). Row shift fraction is the ratio of the lateral shift (Δλ) to the lateral center to center distance (λ). The critical diameter in a DLD array is also heavily influenced by the ratio of the downstream gap (Dx) to the lateral gap (Dy).

DLD separates particles based on their size. Hunag et al. [43] used an arrangement of posts with an offset from the flow direction in a microchannel to introduce the DLD for the first time. The basis of separation in DLD depends on several parameters, including the distance between the posts, the diameter of the posts, and the degree of slope of the posts as depicted in Figure 1. As seen in Figure 1, size-dependent separation of the particle streamlines can be accomplished as it crosses the posts. Two design variables, the gap size (G) between the posts and the angle (α) between the post array and the flow path, determine the separation’s critical diameter (D_c_). Particles smaller than the critical diameter move zigzag between the posts with almost little movement in the y direction, whereas larger-diameter particles bump on the posts and undergo lateral displacement. In other words, small particles follow the flow’s path, but large particles follow the post’s path. The zigzag mode is the first, while the bump or displacement mode is the second. As a result, there is a critical diameter at which the zigzag mode changes to the bump mode. Each row in a DLD array is laterally displaced from the one before it. The critical diameter is determined by the gap values and post shapes.

The crucial variables in a DLD array with circular posts are shown in Figure 1. The row shift fraction, denoted as (ε), is the ratio of the lateral shift (∆λ) to the lateral center-to-center distance (λ),

(1) $$\varepsilon =\frac{∆\lambda }{\lambda }$$

where *λ* is the lateral center-to-center distance which is the sum of the post’s diameter and the lateral gap as illustrated in Figure 1.

(2) $$\lambda =D_{y}+D_{p}$$

Another significant factor in calculating the D_c_ is the downstream to lateral gap ratio and is a metric that has the following definition:

(3) $$\delta =\frac{D_{x}}{D_{y}}$$

where D_x_ and D_y_, respectively, stand for the downstream gap and lateral gap ratio. Inglis assumed that the flow stream between two consecutively spaced posts is partitioned into N flow lanes, where N represents the periodicity of the arrays [66].

(4) $$N=\frac{1}{\varepsilon }$$

By assuming that the fluid flux in each of these lanes is equal (nearest to the post), the width of the first flow lane may be computed. D_c_ is two times the width mentioned earlier and calculated through the method below [66]:

(5) $$\frac{D_{c}}{D_{y}}=1+2\alpha +\frac{1}{2\alpha }$$

where D_c_ is the critical diameter, and *α* is defined as follows:

(6) $$\alpha ={\left[\frac{1}{8}-\frac{\varepsilon }{4}+\sqrt{\frac{\varepsilon }{16}(\varepsilon -1)}\right]}^{\frac{1}{3}}(-\frac{1}{2}-\frac{i\sqrt{3}}{2})$$

The imaginary unit in the equation above is denoted by *i*. Afterward, Davis et al. demonstrated that the D_c_ is underestimated by this computation. They published an empirical formula for D_c_ in DLD devices [79]:

(7) $$\frac{D_{C}}{D_{y}}=1.4\varepsilon^{0.48}$$

Figure 1 shows the mentioned parameters in a DLD device with *n* = 4, demonstrating the segmentation of the flow into *n* (=1 to 4) steams. Each stream passes beneath *n* columns with the n_1_ stream (green) flowing beneath the first column, the n_2_ stream (brown) beneath the second column, and so on. Additionally, it can be determined that the top rows follow the same pattern. It is important to note that the forerunners published the equations mentioned above in the DLD field, which was an important innovation. However, a significant discrepancy exists between the theoretical and practical configurations for various factors. The estimated D_c_ is difficult to attain in the experiment due to non-ideal circumstances, such as finite boundary interfaces in practical devices [80].

### 2.2. Developing Boundary Correction

The DLD array’s internal gap size (G) is fixed at 50 μm. The procedure for creating a wall rectification technique for both the upper and lower walls is described in the following section. The wall-correction technique makes every effort to achieve excellent separation efficiency. It is necessary to know flow behavior, particularly close to channel walls and boundaries, to achieve an effective wall-correction solution.

**Figure 2:**
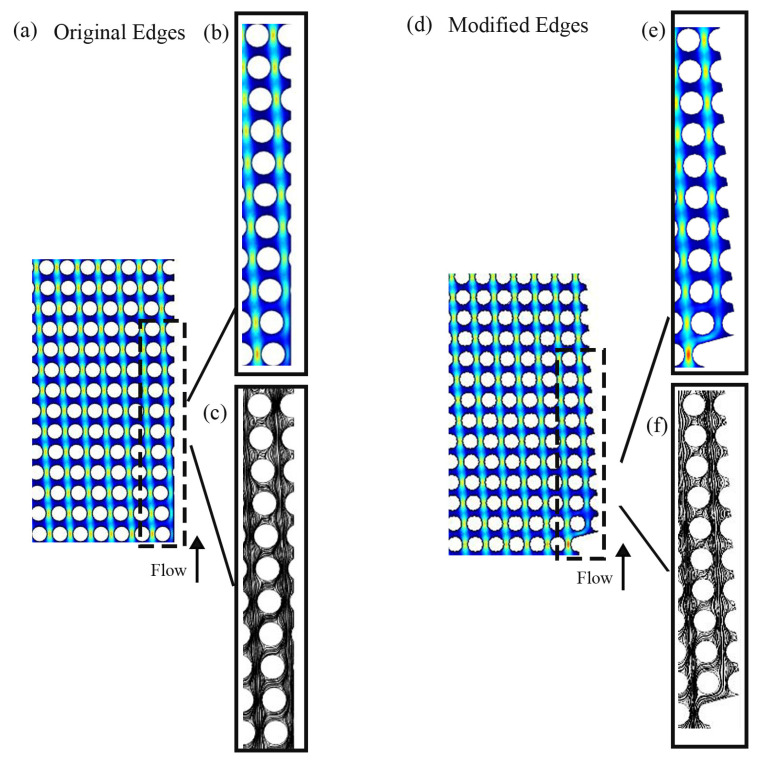
DLD simulations models with (**a**) original and (**d**) modified edges. A closer look at (**b**) the velocity contour, (**c**) the streamline of original edges, (**e**) the velocity contour, and (**f**) the streamline of modified edges. The color scale represents fluid velocity: blue for low-velocity or stationary regions (e.g., near the channel walls), green for intermediate-velocity regions, and red for high-velocity regions (e.g., at the center of the channel in laminar flow).

Many forces, such as drag and lift, influence the subjects flowing in a microchannel [81,82,83,84]. These forces result from shear and normal stresses affecting the particles. Drag forces and lift forces are directed parallel and at the right angle to the main flow direction. The movement of particles inside the microchannel is a function of drag and lift forces. Particle position is caused by the equilibrium between lift and drag forces. The balance of these two forces is disturbed in the unmodified wall boundary, which causes one of the forces to prevail over the other. As shown Figure 2, in the original wall, the microchannel at the boundaries has an irregular shape compared to the middle of the channel. The streamlines are disturbed near the walls, leading to the imbalance of the lift and drag forces. This leads to the fact that some of the particles near the walls do not go through the bump or the zigzag mode and collide with the boundaries and get stuck, thus reducing the separation efficiency. If, by modifying the walls, the streamlines and the velocity distribution can be regularized, then the balance between the forces can be established. In fact, the streamlines that show the movement of fluid molecules move consistently in the channel with the modified wall, and both the streamlines and velocity distribution are correctly formed. This establishes the balance between drag and lift forces, and the cells are positioned at a suitable distance from the wall and move regularly.

We consider a particle-concentrating apparatus with two inputs, as seen in Figure 1, where particles enter the microchannel, and those larger than the critical size move along the slope of the channel. Without careful engineering, neither the bottom nor the top edge can laterally shift particles. This results in low concentrations and residual particles that remain in the channel. The separation process is based on a minimal fluid flux, a streamline, to pass over the upper side of each post. The streamline’s width defines the critical size of the particles. For effective separation, a consistent D_c_ is necessary. The boundaries significantly impact this value. The first and last gaps in each row must be appropriately controlled.

The design in Figure 2 was used to fabricate the microchannel in this report. The microchannels were designed using a CAD software (AutoCAD Version 2023) and fabricated by conventional photolithography. The SU-8 photoresist was directly used to fabricate the master molds on silicon with a height of approximately 40 μm. Afterwards, the replications of PDMS (Sylgard 184, Dow Corning, Midland, MI, USA), USA were created and bonded to glass slides. The cells’ response time is captured using a camera under an inverted microscope (Basler ace acA800-510uc, Basler, Ahrensburg, Germany). The cancer cell lines move as predicted from wall to wall and condense into a single column at the right-side boundary.

**Figure 3:**
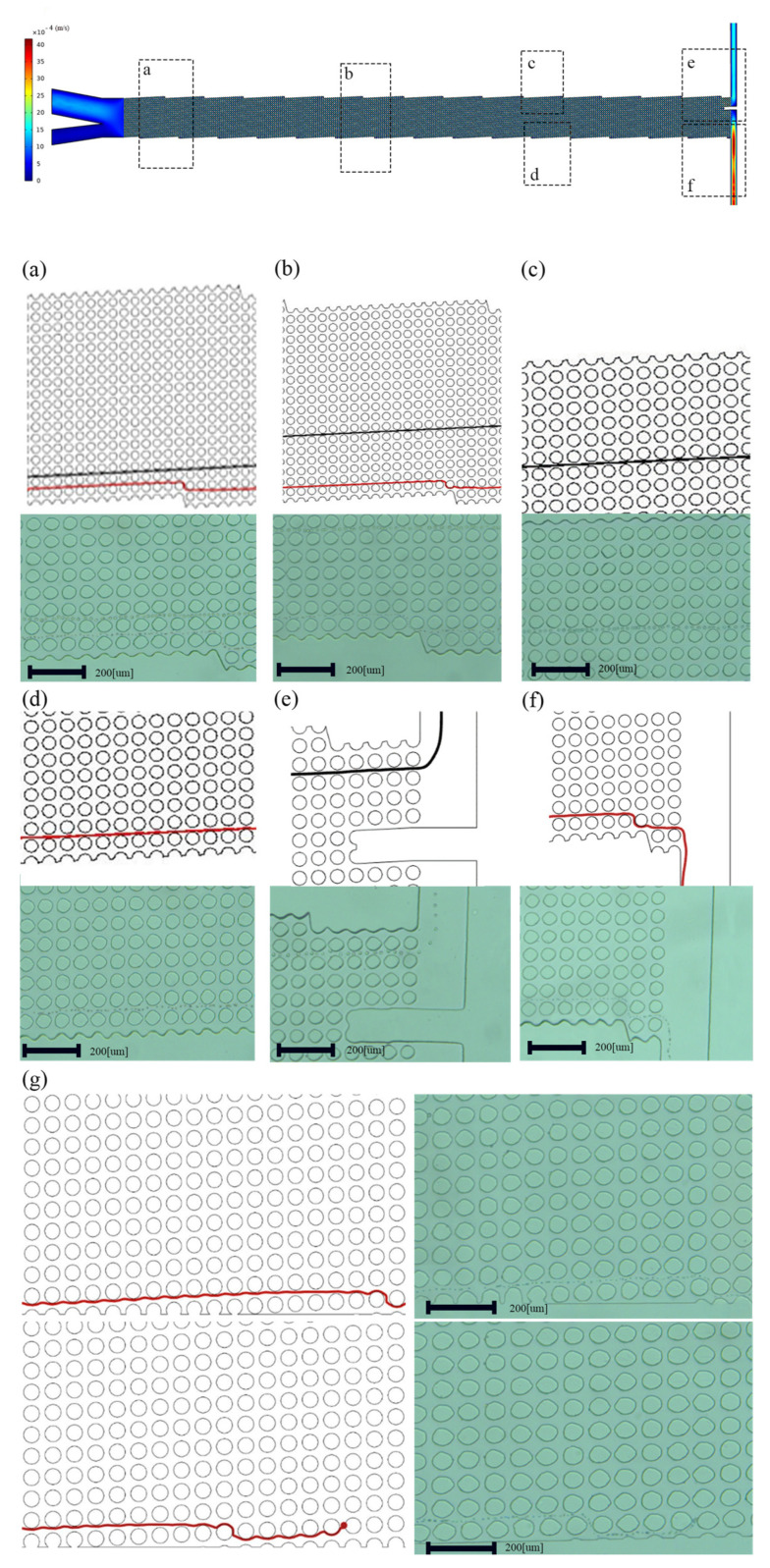
Both fluid simulations and experiments showing (**a**–**f**) modified edges and (**g**) original edges. A closer examination reveals how the device’s boundary affects particle separation and clogging in the channel.

This design is validated by fabrication and testing with cancer cell lines. A microfluidic channel was used for comparing DLD arrays. Figure 3a–f has vertical or modified edges, while Figure 3g has unmodified edges.

**Figure 4:**
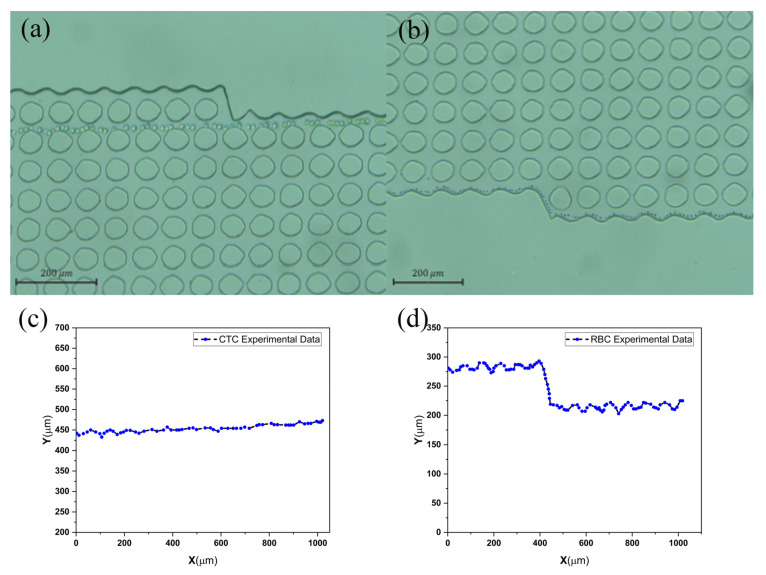
(**a**) CTC and (**b**) blood cell separation image and the movement path of two particles in (**c**) bump and (**d**) zigzag mode.

The arrays are 1 cm wide and 3.8 cm long, and they contain 310 columns and 20 rows. The gap (G) between the posts and the post diameter are both 50 μm, and the row shift fraction ε is 0.04, so the array replicates itself every 20 rows. An up-close examination of the edges is displayed in Figure 4. Tweaking the edges enhances both the efficiency near these edges and shows that the fabricated microchannel significantly improves the overall effectiveness of the array. Before rectifying, the boundaries influence the D_c_ at the center of the array, based on the design of the microchannels. The qualitative impact of changes in D_c_ resembles the effects of particle size dispersion.

A more precise modeling approach could lead to further advancements. Following a particle through a series of arrays with a range of critical sizes, the migration angle, also known as the slope, is computed. If a particle exceeds the D_c_, it experiences displacement due to the row shift and is elevated by the array period. A particle moves down by the array period but not laterally if it is smaller than the critical size. A migration slope is created by repeating this.

### 2.3. Fabrication

**Figure 5:**
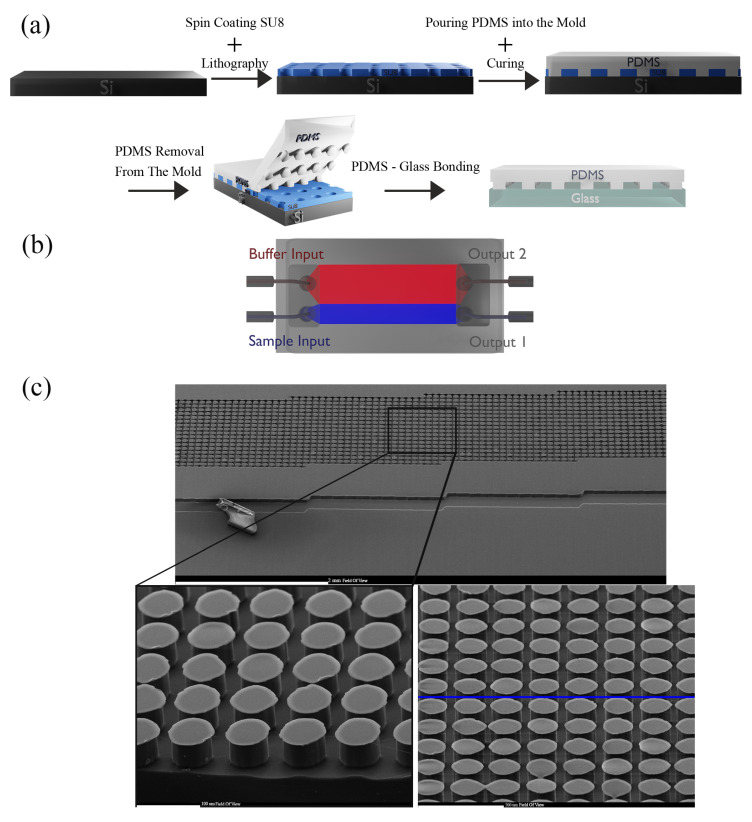
(**a**) The fabrication process of the DLD microfluidic channel. (**b**) The final device fabricated in PDMS using soft lithography. (**c**) SEM of the independent DLD sorter’s construction showing the circular posts used to study the kinetics of particle/cell migration.

The fabrication process of the DLD sorter is as follows in Figure 5a. An array of circular posts with a fixed diameter (D_P_) and gap (G) of 50 µm was employed in the design of the DLD device. A process was established to obtain a critical size (D_C_) set nearly at the target range of ≈15 µm. The row shift (∆λ) of the chosen D_C_ was 4 µm. With a post height of 40 µm, the aspect ratio of the DLD device was maintained at 1.25. The boundary corrections for each array period followed the previously reported empirical relations. The input sample was contained within the apparatus by a sheath flow, which maximized sample contact with the DLD post array and allowed for a shift of particles larger than DC (Figure 1). The device for DLD separation was fabricated using standard photolithography methods. The RCA Standard Cleaning technique is used primarily to clean the substrate. The silicon wafer was patterned with a mask and UV light covered with a SU-8 photoresist (2050, Kayaku, Westborough, MA, USA).

The master mold was fabricated and hard-baked to improve mechanical resistance and minimize surface flaws and then utilized for micro molding. The well-blended PDMS mixture was poured into the SU-8 mold on Si with a base-to-curing agent weight proportion of 10:1. The PDMS with channel geometry was peeled from the mold after curing at 75 °C for 30 min. With a biopsy punch, the inlets and outlets were punched. Glass and PDMS were contacted to bond after activation with oxygen plasma. The DLD sorter was designed to have 50 μm-diameter circular posts. The gap distance (G) was intended to be 50 µm, and the boundary interface was modified to reduce the unsteady flow in the parts close to the boundaries. Figure 5a,b show the fabrication process of our design and the schematic, and Figure 5c depicts the scanning electron microscopy (SEM) of the circular posts. The specifications of the DLD sorter channel are presented in Table 1.

### 2.4. Cell Line Sample Preparation

The PC-3 cells were cultured in RPMI-1640 medium supplemented with 10% fetal bovine serum (FBS), streptomycin (100 Ig/mL) (Gibco BRL; Thermo Fisher Scientific, Waltham, MA, USA), and penicillin (100 IU/mL). The cells, in the exponential growth phase with approximately 70–80% confluency, were cultured for experimental purposes in a humidified atmosphere using a 5% CO_2_ incubator at 37 °C [85].

## 3. Results and Discussion

### 3.1. Experimental Setups

Phosphate-Buffered Saline (1 × PBS, Dulbecco, Sigma-Aldrich, St. Louis, MO, USA) solution was pumped into the microchannel before usage to reduce cell adhesion and clogging of the channel structure. A high-speed camera (Basler ace acA800-510uc) with 750 frames per second was used to record the particle/cell movements in the microchannel, while the process was carried out in the bright-field observation mode. The composite images that show the distributions of particles and cells during a specific period were made by processing the collected image frames using ImageJ Ver.1.54 e. Optimizing the geometry of the channels near the boundaries makes the streamlines and velocity contours completely regular as in the middle of the channel, making the cells pass through the side of the wall and exit the outlet without any clogging. We first simulated two devices with different boundaries, designed to concentrate particles. In the device with the modified boundary, particles approach the device’s lower section, and as seen in Figure 3, particles exceed D_c_ flow at an angle of θ to the fluid flow. In an ideal situation, particles larger than D_c_ would accumulate along the upper wall, covering a distance determined by the ratio of the array width to the tangent of the migration angle θ. Prostate cancer cell lines with a size of 25 µm are larger than D_c_ and concentrate above the wall. WBCs with a diameter smaller than the D_c_ move towards the bottom of the wall in a zigzag mode. On the other hand, in the device with no boundary corrections and careful design, neither the upper nor lower boundaries effectively initiate the lateral displacement of particles, resulting in inadequate concentrations as well as residual particles that were left in the solution, as in Figure 3g.

**Figure 6:**
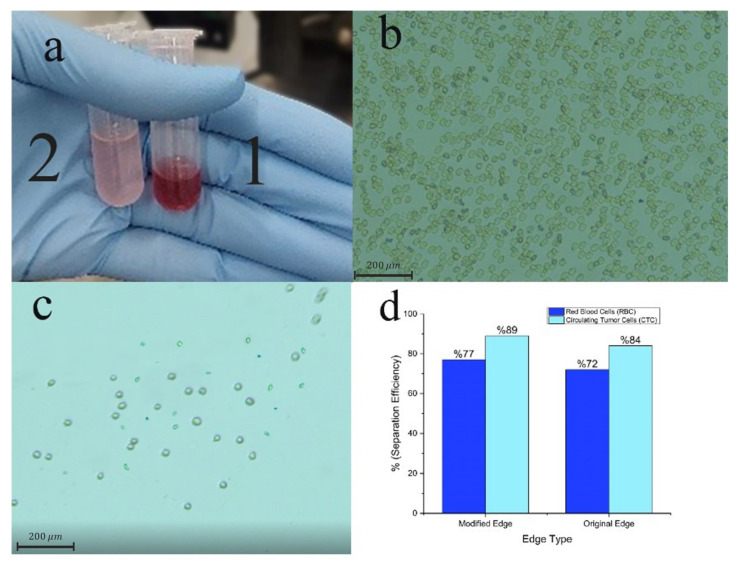
(**a**) Image of the acquired samples after experimentation aimed at separating rare tumor cells from the blood. Microtube 1 is collected from the RBC outlet and Microtube 2 is collected from the CTC outlet. (**b**,**c**) Microscopic visuals of the samples were gathered from both outlets. (**d**) Effectiveness in separating blood cells and tumor cells at each outlet.

Two-dimensional fluid simulations first verify this approach. Using COMSOL MULTIPHYSICS^®^ 5.6, two DLD arrays with comparable structures yet distinct boundaries were examined and contrasted. Then, the devices were fabricated using soft photolithography. Figure 3a–f have edges that are modified in accordance with the principles of the middle of the channel, while Figure 3g has unmodified edges. Modifying the edges enhances efficiency in the proximity of these boundaries and, simultaneously, demonstrates an overall improvement in effectiveness across the DLD array. Additionally, flow adjustment at the boundaries has resulted in a D_c_ that is more consistent, as evident from the classes of particles in Figure 6.

### 3.2. Tumor Cell Separation Characterization

The efficiency of our DLD sorter in separating cells was assessed. We used it to separate rare tumor cells from the blood. The blood samples containing a cellular concentration of 1 × 10^9^ counts/mL was injected with 1 × 10^6^ tumor cells/mL and directed into the DLD separator at a sample flow rate of 100 μL/min and a sheath flow rate of 120 μL/min. Figure 6a displays an image of the samples that were taken from both outlets. The sampled microscopic images taken in the bright field observing mode are shown in Figure 6b,c, and the efficiency of the separation in Figure 6d. Following the separation phase, it was discovered that a significant amount of blood cells could be extracted from outlet 1, giving the gathered sample a reddish appearance. The percentage of the extracted blood cells as a whole is as high as 77%, and no tumor cells were found in the analyzed windows of the samples taken out of this outlet. By observing the microscopic images of target samples, only a limited number of blood cells are noticed near the target tumor cell.

The extra 5% efficiency gained with the boundary correction in the DLD separator is important. Tracking the quantity of circulating tumor cells (CTCs) in a patient’s bodily fluids can offer valuable insights. Improving the reliability of CTC detection devices even by 5% is crucial, since it greatly improves the accuracy of diagnosis, which results in the earlier detection of cancer and the more precise monitoring of disease progression. This enhancement results in more effective treatment adjustments, subsequently improving therapeutic results and diminishing the total burden of cancer treatment.

Moreover, a cell viability test was conducted to assess if the shear stress occurring during the interaction between cells and posts might lead to the death of target tumor cells. The separated tumor cells were subsequently re-cultured for a period of 48 h. Figure S5 illustrates the microscopic images of the re-cultured cells, confirming that our sorter has little impact on cell viability. Furthermore, the separated cells are capable of being re-cultured. Hence, our DLD sorter has the capacity to separate CTCs without the need for labeling, ensuring both high viability and high purity [86].

## 4. Conclusions

In this study, two modes, with modified and unmodified boundaries, of a DLD separator for label-free cell separation were investigated. However, the boundaries should not be such that they disrupt the flow lines from the state between the posts away from the wall. Boundary correction techniques have been used to maximize the precision and efficiency of DLD devices and further expand the original idea. When DLD arrays are used in nano-channels, micro-channels, and specific uses, wall-correction techniques become necessary. In this design, we have developed a DLD sorter for precise, continuous, and label-free cell separation with boundary correction (BC) introduced to eliminate boundary interface-related disharmonies that might adversely affect separating cells or particles and reduce the efficiency of separation. This method prevents particle clogging of the channel walls during the path of the DLD microchannel, and the separation is performed with higher efficiency. Despite managing a sample with a high amount of large tumor cells, no clogs were observed in the DLD array. Our DLD sorter has the potential to find widespread application in the future for efficiently separating multiple types of cells based on size, without the need for labels. The characterization results show that more than 89% separation of 25 μm and 7 μm particles is achieved.

## Figures and Tables

**Table 1:** Detailed specifications of the DLD sorter channel.

Dimension	Value
Post diameter (D_post_)	50 [µm]
Gap between posts in a row (g)	50 [µm]
Gap between rows (Dy)	g
(λ)	G + D_post_
Post shifts between nearby rows (Δλ)	4 [µm]
Period (N)	λ/Δλ
Post shift ratio (ε)	1/N
Critical diameter (D_c_)	1.4 × g × (ε)^0.48^

## Data Availability

All data supporting reported results are available on request from the authors.

## Supplementary Materials

The following supporting information can be downloaded at: https://www.mdpi.com/article/10.3390/bios14100466/s1, Supporting Pdf: Supporting information.pdf; Video S1: Supplementary video S1.mp4, Video S2: Supplementary video S2.mp4, Video S3: Supplementary video S3.mp4, Video S4: Supplementary video S4.mp4.
