# Supplementary material for: Effective Boundary Correction for Deterministic Lateral Displacement Microchannels to Improve Cell Separation: A Numerical and Experimental Study
Source: Biosensors (Basel). 2024 Sep 29;14(10):466. doi: 10.3390/bios14100466 (PMC11506467; doi:10.3390/bios14100466)
Supplement: Supplementary file 1 [file biosensors-14-00466-s001.zip › Supporting information.pdf]

Supporting information

Effective boundary correction for deterministic lateral displacement microchannels to improve cell separation: Numerical and experimental study

Shaghayegh Mirhosseini[1,4], Mohammad Mahdi Eskandari Sani[2,5], Aryanaz Faghih Nasiri[1], Fatemeh Khatami[3], Akram Mirzaei[3], Majid Badieirostami[1], Seyed Mohammad Kazem Aghamir[3]**, Mohammadreza Kolahdouz[1]*

1. School of Electrical and Computer Engineering, College of Engineering, University of Tehran, Tehran, Iran
2. School of Mechanical Engineering, College of Engineering, University of Tehran, Tehran, Iran
3. Urology Research Center, Tehran University of Medical Sciences, Tehran, Iran
4. Department of Electrical and Computer Engineering, University of Virginia, Charlottesville, VA 22908, USA
5. Department of Bioengineering, University of Pittsburgh, Pittsburgh, Pennsylvania, USA

*Email: kolahdouz@ut.ac.ir

**Email: mkaghamir@tums.ac.ir

Figure S1. Structure of the separate DLD sorter with circular posts.

Figure S2. Distributions of tumor cells and blood cells across the channel width at the outlet of the DLD sorter

Figure S3. Distributions of tumor cells and blood cells across the boundary of the channel width at the outlet of the DLD sorter.

Figure S4. Microscopic images of the samples collected from two outlets (the bottom outlet related to the blood cells and the upper outlet related to tumor cells) under bright-field mode.

Figure S5. Microscopic images of the separated tumor cells which were re-cultured for 48 hours.

Figure S6. Scanning Electron Microscopy of the DLD sorter when the posts are not fabricated properly.

Table S1. Dimensions of our DLD sorter channel.

Section S1. The fabrication process of our DLD sorter.

Supplementary video S1. Video illustrating the bump mode movement of tumor cells across the channel of the DLD sorter.

Supplementary video S2. Video illustrating the zigzag mode movement of blood cells across the channel of the DLD sorter.

Supplementary video S3. Video illustrating the movement of tumor cells across the boundary of the channel of the DLD sorter.

Supplementary video S4. Video illustrating movement of blood cells across the boundary of the channel of the DLD sorter.

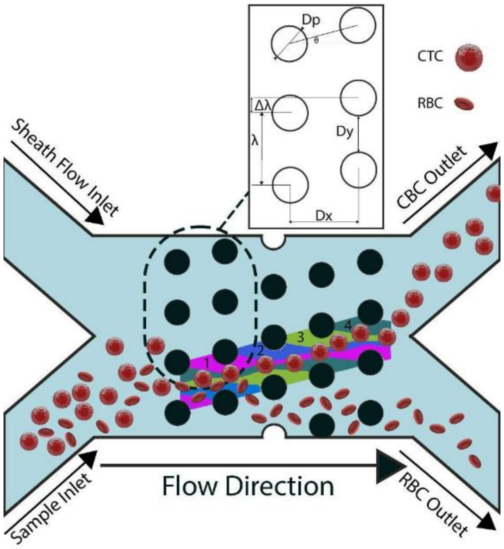

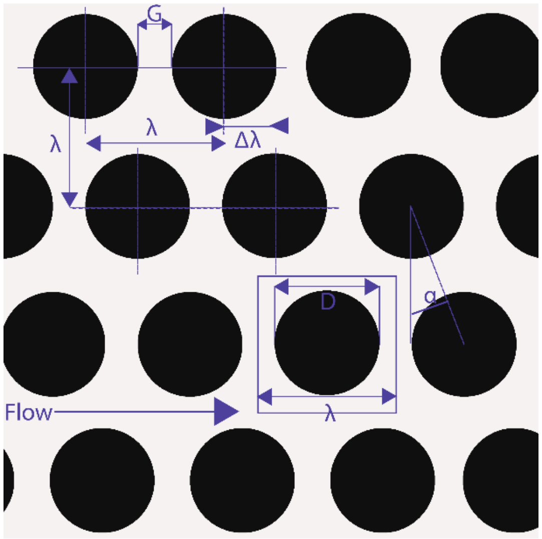

Figure S1 illustrates the configuration of an independent DLD sorter featuring circular posts, designed to investigate the dynamics of particle/cell migration. This sorter incorporates a two-inlet system, and its geometry is identical to the one utilized in our DLD sorter. The independent DLD sorter utilizes a sheath flow to restrict the sample flow to the bottom of the channel region. The DLD array in this design comprises 16 repeated units of post arrays. The separation of particle streamlines based on size is evident as they traverse the posts. The critical diameter (Dc) of this separation is determined by two design variables: the gap size (G) between the posts and the angle (α) formed by the post array and the flow path. Particles smaller than the critical diameter exhibit a zigzag movement between the posts with minimal displacement in the y-direction, while larger particles collide with the posts, experiencing lateral displacement. Essentially, small

particles follow the flow path, while large particles follow the path defined by the posts. The zigzag mode represents the first, and the bump or displacement mode is the second. Consequently, a critical diameter exists at which the transition from the zigzag mode to the bump mode occurs.

In a DLD array with circular posts, as illustrated in Figure S1, key variables include the row shift fraction (ε), which is the ratio of the lateral shift (Δλ) to the lateral center-to-center distance (λ). Here, λ is the lateral center-to-center distance, calculated as the sum of the post's diameter and the lateral gap. Another crucial factor in determining Dc is the downstream to lateral gap ratio, where Dx and Dy represent the downstream gap and lateral gap ratio, respectively.

The crucial parameters of a DLD device with n = 4 are depicted in Figure 1, showcasing the segmentation of the flow into n (= 1 to 4) streams. Each stream passes beneath n columns, with the n1 stream (pink) flowing beneath the first column, the n2 stream (blue) beneath the second column, and so forth.

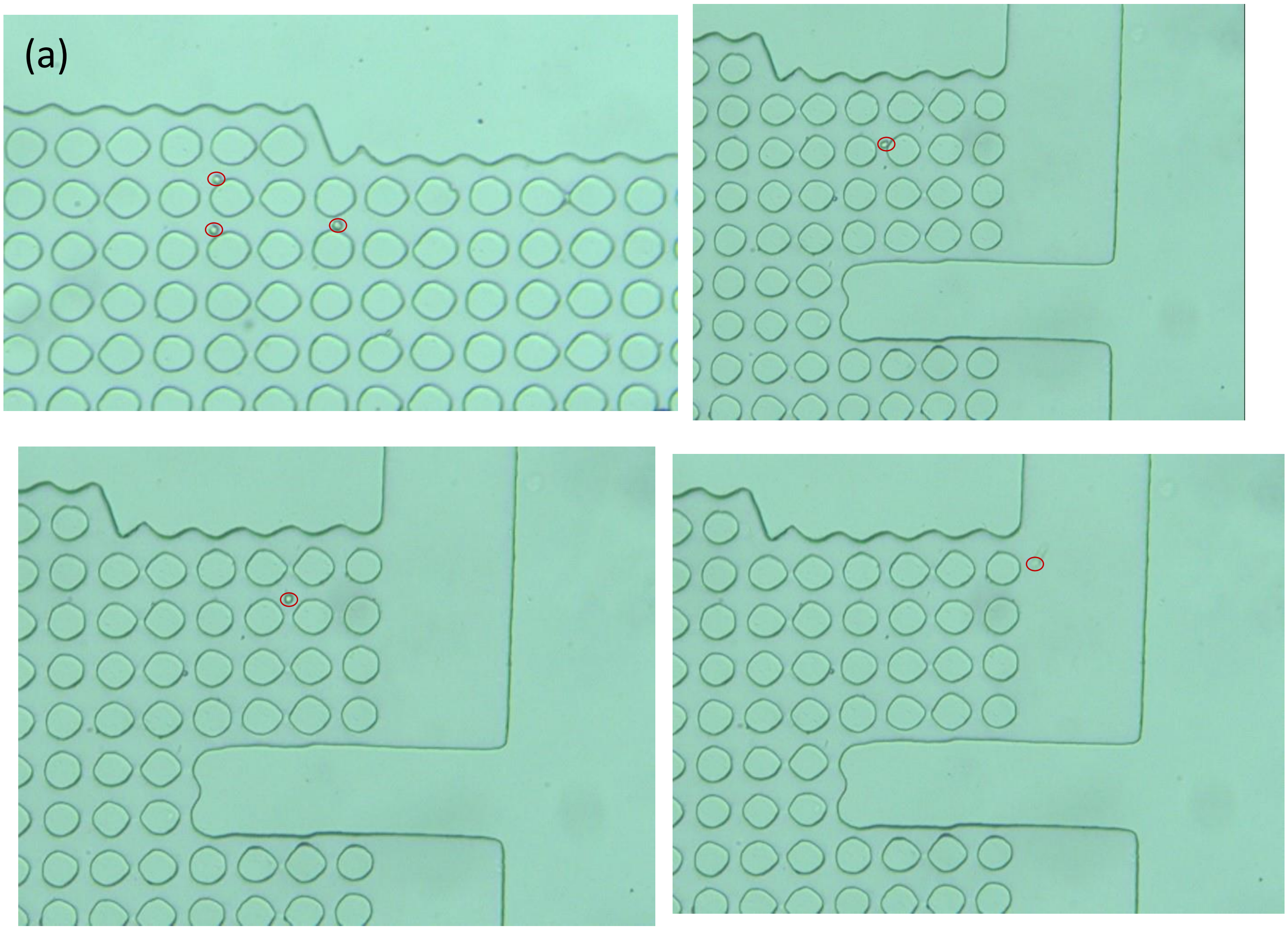

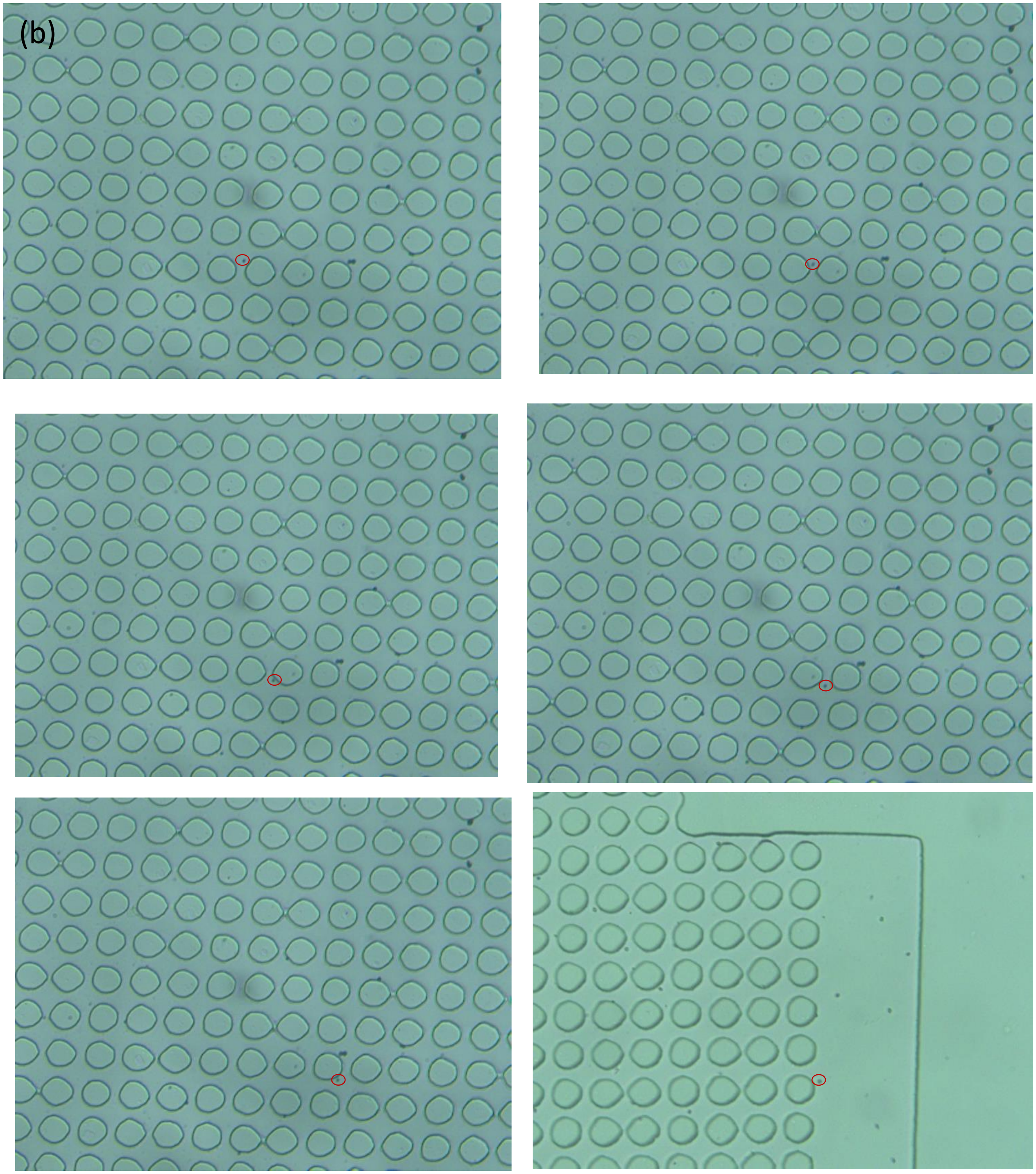
(b)

Figure S2 depicts the spatial distributions of tumor cells (a) and blood cells (b) near the outlet of the independent DLD sorter, specifically across the channel width. These observations were made under flow conditions of approximately 100µL/min. It is noteworthy that the concentrations of tumor cells and blood cells in the conducted experiments were deliberately set at 103 counts/mL and 107 counts/mL, respectively. The controlled concentrations provide a basis for studying and understanding the behavior and interactions of tumor cells and blood cells within the separate DLD sorter under the specified flow conditions.

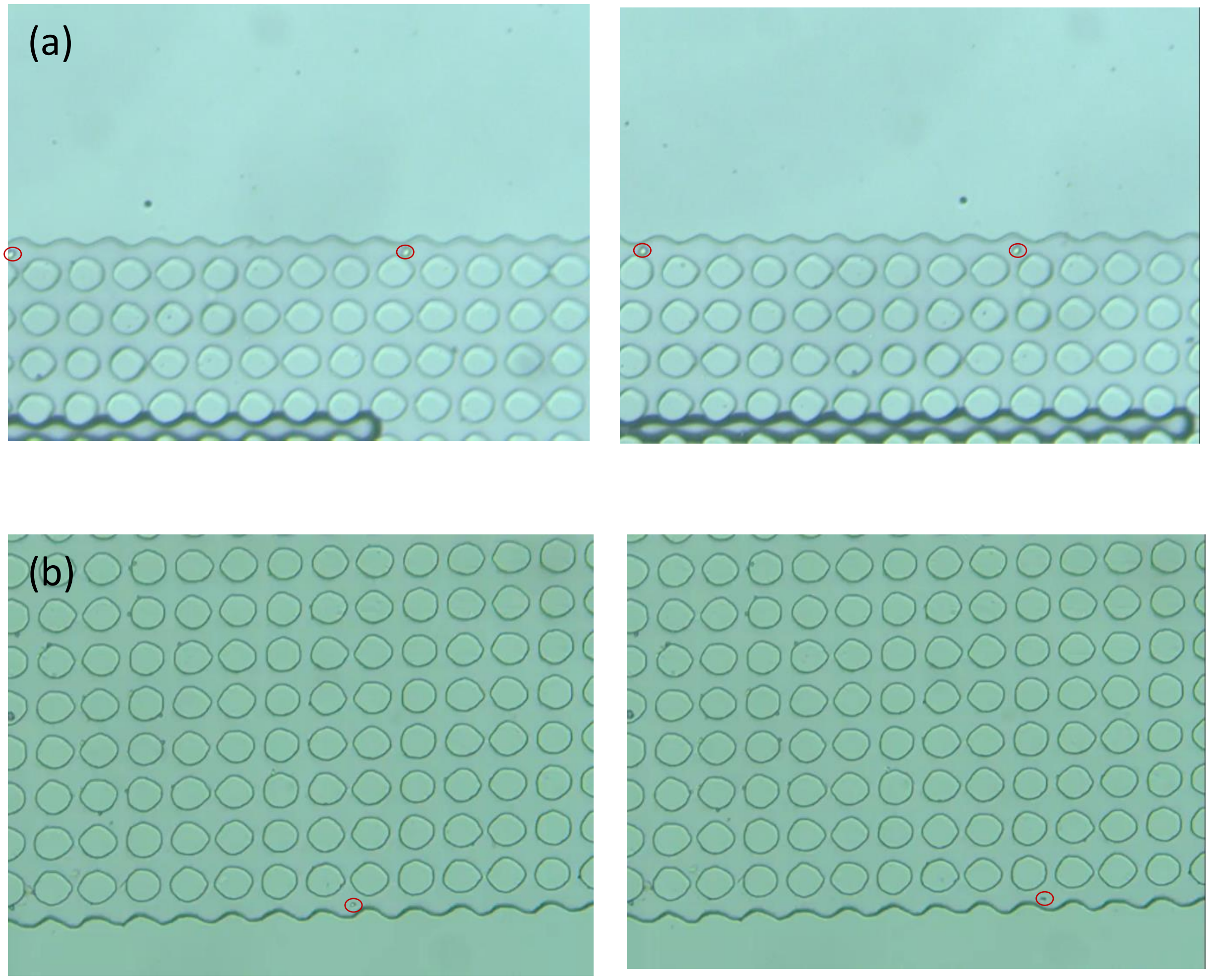

Figure S3 depicts the investigation of dispersion patterns of tumor cells (a) and blood cells (b) along the channel boundary in an individual deterministic lateral displacement (DLD) sorter, where the flow rates are approximately 100µL/min. This exploration aims to analyze the spatial distribution of both tumor and blood cells as they cross the boundary while

traversing the channel, providing valuable insights into the sorting dynamics within the DLD device under these boundary correction conditions.

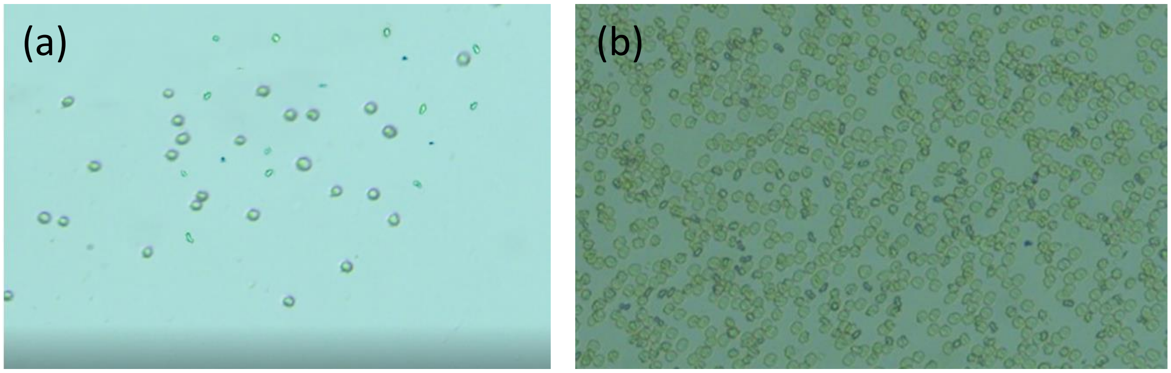

Figure S4. Microscopic images of the samples, (a) tumor cells and (b) blood cells, collected from outlets under bright-field mode.

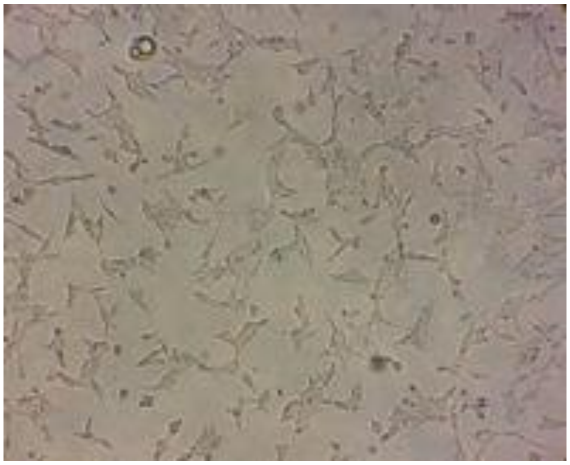

Figure S5. Microscopic images of the separated tumor cells which were re-cultured for 48 hours.

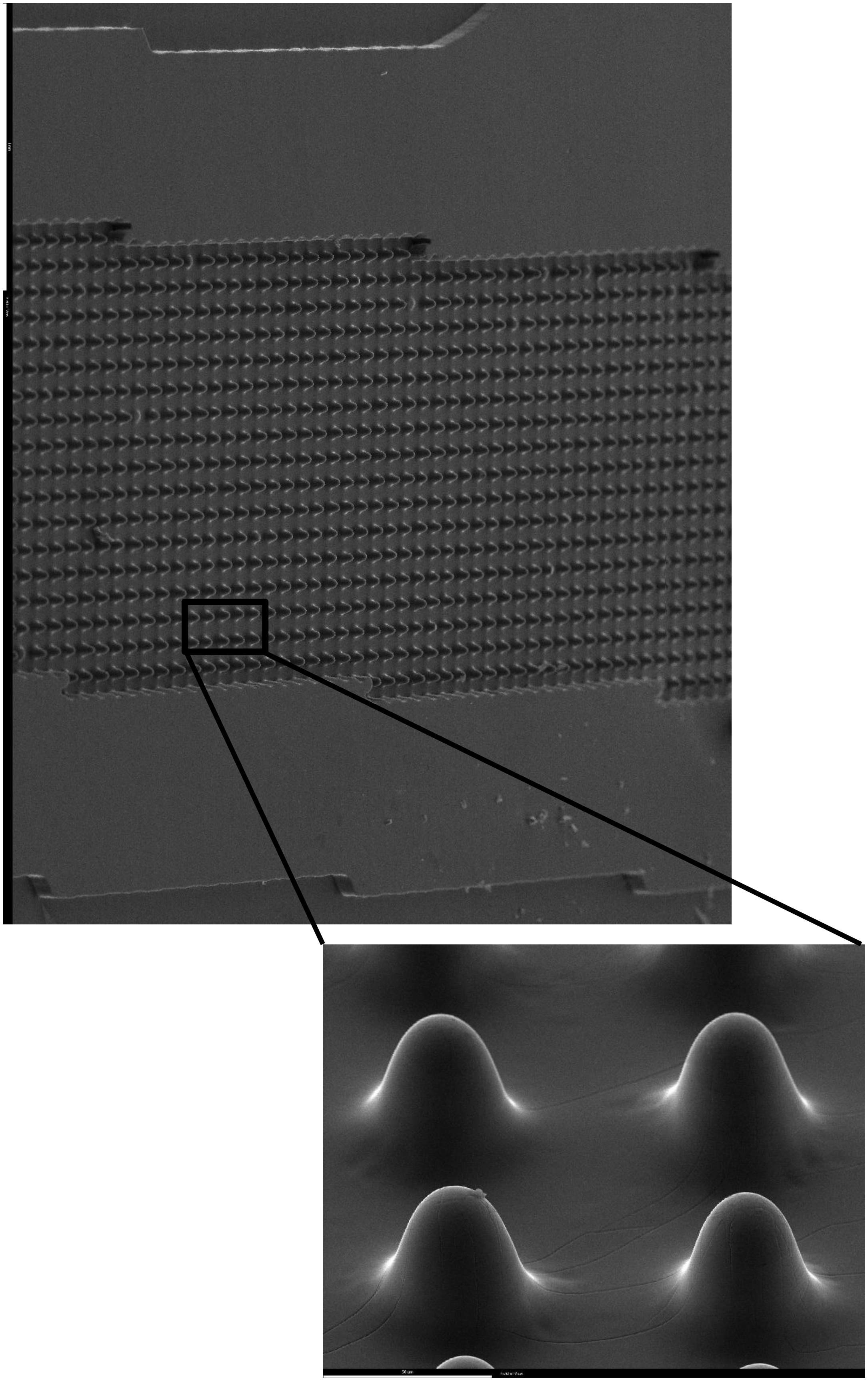

Figure S6. Scanning Electron Microscopy of the DLD sorter when the posts are not fabricated properly.

Table S1. Dimensions of the employed in our DLD sorter.

**Dimension**	**Value**
Post diameters (D_{post})	50[um]
Gap between posts in a row (g)	50[um]
Gap between rows (Dy)	g
(λ)	$G + D_{post}$
Post shifts between nearby rows (Δλ)	4[um]
Period (N)	λ/Δλ
Post shift ratio (ε)	1/N
Critical diameter (D_c)	1.4*g*(ε)^0.48

Section S1. Description of the DLD sorter fabrication process.

In summary, the process involved coating a silicon wafer with a layer of SU-8 photoresist (2050, Microchem), followed by UV light patterning using a photomask. Subsequent steps included developing and hard baking, resulting in the fabrication of a master mold used for micro molding. A PDMS mixture (Sylgard 184) with a base-to-curing agent weight ratio of 10:1 was then cast onto the master mold. After curing at 75 °C for 45 minutes, the PDMS block with channel geometries was removed from the master mold and cut into smaller pieces. Inlet and outlet orifices were punched, and the PDMS block's channel surface underwent treatment using an oxygen plasma cleaner. Finally, the PDMS block was bonded with oxygen plasma to a clean glass slide to seal the channel.

Supplementary video S1. Video illustrating the bump mode movement of tumor cells cross the channel of the DLD sorter.

Supplementary video S2. Video illustrating the zigzag mode movement of blood cells cross the channel of the DLD sorter.

Supplementary video S3. Video illustrating the movement of tumor cells cross the boundary of the channel of the DLD sorter.

Supplementary video S4. Video illustrating movement of blood cells cross the boundary of the channel of the DLD sorter.
